# Anti-tumor vaccine efficacy depends on adjuvant type and associates with induced IgG subclass and glycosylation profiles

**DOI:** 10.1186/s40164-025-00708-6

**Published:** 2025-10-06

**Authors:** Selina Lehrian, Anna Wasynczuk, Janina Petry, Melanie Guderian, Jan Nouta, Jana Sophia Buhre, Hanna B. Lunding, Philipp Köcher, Hannah Franziska Schumacher, Lara Dühring, Kathleen Kurwahn, Kristina Manzhula, Rudolf Manz, Yannic C. Bartsch, Manfred Wuhrer, Marc Ehlers

**Affiliations:** 1 Laboratory of Immunology, Institute of Nutritional Medicine, University of Lübeck and University Hospital Schleswig-Holstein, Ratzeburger Allee 160, 23562 Lübeck, Germany; 2 Center for Proteomics and Metabolomics, Leiden University Medical Center, Leiden, The Netherlands; 3 Laboratory of Anti-Viral Antibody-Omics, TWINCORE – Institute for Experimental and Clinical Infection Research, Helmholtz Center for Infection Research (HZI) and Medical School Hannover (MHH), Hannover, Germany; 4 Institute of Systemic Inflammation Research, University of Lübeck and University Hospital Schleswig-Holstein, Lübeck, Germany; 5 German Center for Lung Research (DZL), Lübeck, Germany

**Keywords:** Tumor, Vaccination, Antibody, IgG subclass, IgG glycosylation, IgG fucosylation, NK-cell, Neutrophil

## Abstract

Vaccination with tumor-(neo) antigen plus adjuvant is emerging as a promising cancer-therapy. However, as different adjuvants induce distinct immune cell and antibody (Ab) responses, selecting the right adjuvants remains challenging. Here, we evaluated the following vaccine adjuvants to promote protection against tumor-growth in mice and correlated IgG subclass and Fc *N*-glycosylation responses: Alum; the toll-like receptor activators Poly(I:C) and MPLA; Alum-Poly(I:C); and the more inflammatory water-in-oil adjuvants Montanide, IFA, CFA, and *M.tb.*-enriched (e)CFA. While Alum and Montanide failed to protect, MPLA and IFA tended to protect, and Poly(I:C), Alum-Poly(I:C), CFA, and eCFA significantly protected against tumor-growth. Across all adjuvants, tumor-protection correlated with the induction of highly activating IgG2(c/b) Abs and afucosylated (F0) IgG1 Abs, the latter showing up to 5% abundance. While all adjuvants transiently induced IgG1 F0 following initial immunization, Poly(I:C)- and eCFA-induced memory responses also generated IgG1 F0 after repeated antigen-exposure without adjuvants. Additionally, Poly(I:C)-induced tumor-protection was associated with high IgG2c/IgG1 ratios, high levels of IgG galactosylation and sialylation, and IFNγ-producing CD8 + Tc1-cells. Conversely, Ova-eCFA-induced tumor-protection was additionally associated with high levels of IgG across all subclasses, but low levels of galactosylation and sialylation, and CD8 + Tc17- and CD4 + Th17-cells. Accordingly, tumor protecting adjuvants may induce common but also different protecting programs. A tumor-antigen-specific IgG2a monoclonal (m)Ab protected against tumor-growth in both its de-galactosylated and galactosylated plus sialylated forms, suggesting common and possibly distinct protective mechanisms. Tumor-protection via serum transfer from Poly(I:C)-immunized mice depended more on NK-cells, whereas eCFA-induced and non-sialylated/non-galactosylated mAbs promoted neutrophil activation. These findings may help to improve tumor vaccination protocols.

**Graphical abstract:**

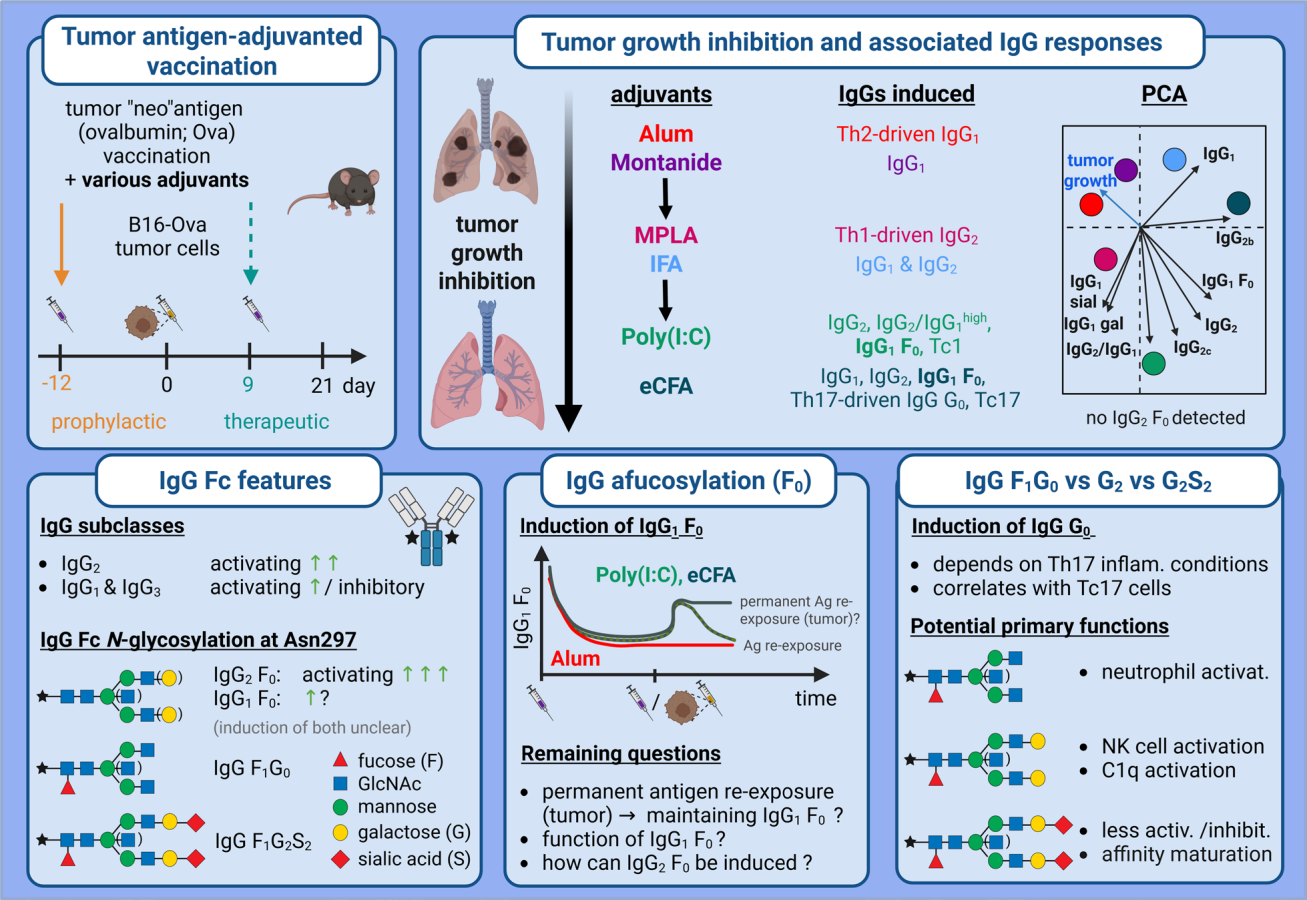

**Supplementary Information:**

The online version contains supplementary material available at 10.1186/s40164-025-00708-6.

## To the Editor,

**Fig. 1:**
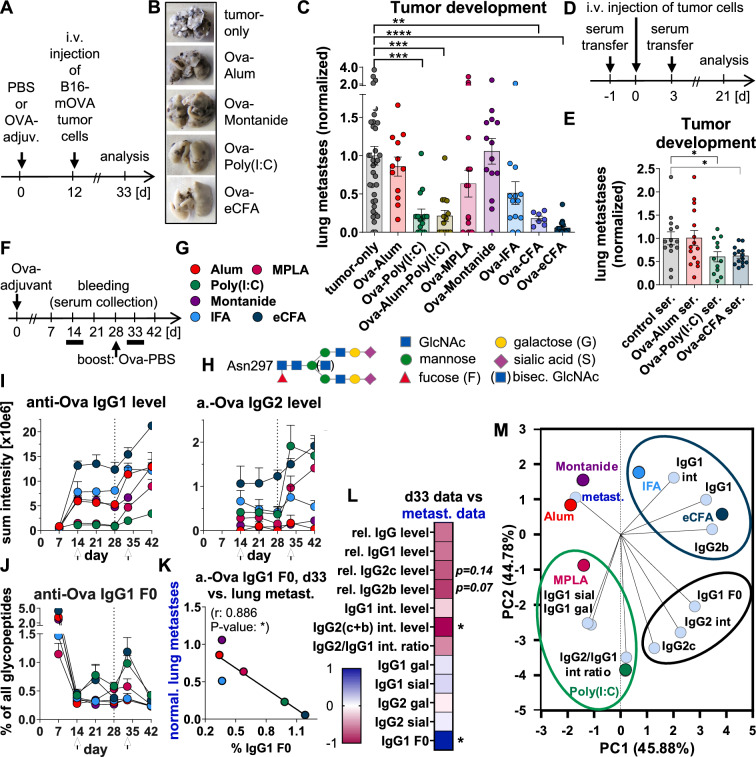
Vaccine-adjuvant-induced protection against tumor-growth and its correlation with antibody responses. **A**–**C** Vaccine-adjuvant-induced protection against tumor-growth. (**A**) Experimental design: C57BL/6 mice were immunized prophylactically (i.p.) with Ova plus different adjuvants on day 0, injected (i.v.) with B16-mOVA melanoma cells on day 12, and analyzed 21 days later on day 33. (**B**) Representative images of fixed lungs with metastases. (**C**) Normalized number of lung surface metastases from five combined experiments. Statistics: One-way ANOVA with Dunett's multiple comparisons test. **D-E** Serum transfer from Ova-adjuvant-immunized mice to other B16-mOVA-injected mice. (**D**) Experimental design: serum (ser.) was collected from mice 12 days after immunization with Ova-Alum, -Poly(I:C) or -eCFA or from non-immunized mice (control ser.) and transferred to other mice on days -1 before and 3 after B16-mOVA-cell injection. (**E**) Normalized number of lung metastases from multiple experiments on day 21. Statistics: unpaired t-tests. **F**–**J** IgG subclass levels and Fc glycosylation patterns after Ova-adjuvant immunization. (**F**) Experimental design: mice were immunized with Ova plus different adjuvants, boosted on day 28 with Ova without adjuvant and analyzed on the indicated days (n = 5 per group) [9]. (**G**) Color code of the adjuvants studied. (**H**) Schematic representation of the Fc-glycosylation pattern G2S2(B)F. (**I**) Mean levels of anti-Ova-IgG1 and -IgG2 (both IgG2c and IgG2b) determined by summing the intensities (absolute numbers) of all identified anti-Ova-IgG1 and -IgG2 glycopeptides, respectively, measured by nLC-MS (not described in the prior publication [9]). (**J**) Percentage of anti-Ova-IgG1 afucosylated (F0) from all anti-Ova-IgG1 glycopeptides (not described in the prior publication [9]). Anti-Ova IgG2 F0 and IgG3 F0 were not detected. **K**–**M** Correlation of the day 33 antibody data from the immunization experiment with the normalized (normal.) number of metastases (metast.) from the tumor-vaccination experiment shown in (**C**). (**K**) Correlation of anti-Ova-IgG1 F0 with normalized numbers of metastases. Pearson's correlation (r)- and p-values are shown. (**L**) Heat map of Pearson correlation-values (r) between relative (rel.) anti-Ova-IgG or -IgG subclass levels measured by ELISA as well as summed intensity levels, ratios and glycosylation patterns measured by nLC-MS and normalized numbers of lung metastases. Selected p-values are shown. (**M**) Principal component analysis (PCA) of the Ab data and normalized numbers of lung metastasis (loadings), showing each loading and the score for each adjuvant. Results of interest are outlined; see text. *p < 0.05, **p < 0.01, ***p < 0.001, ****p < 0.0001

Tumor-(neo)antigen-specific vaccination with adjuvants is an innovative tumor-therapy [1]. However, the optimal adjuvants for inducing effective T- and B-cell and IgG Ab responses remain unclear. IgG effects depend on subclass levels, ratios and Fc *N*-glycosylation patterns. Human IgG1 and IgG3, and mouse IgG2a/c and IgG2b, are potent activators of Fcγ receptors and C1q, whereas human IgG2 and IgG4, and mouse IgG1 and IgG3, have less activating and also inhibitory properties [2, 3]. The glycan core structure at IgG-Fc-Asn297 can be modified with fucose, bisecting *N*-acetylglucosamine (GlcNAc), galactoses and sialic acids (Fig. 1). Afucosylation (F0) strengthens the anti-tumor effect of human IgG1 and IgG3 via FcγRIII, and of mouse IgG2(a/c and b) via FcγRIV [2, 4–6]. Notably, a 1% variation in F0 can already significantly impact effectiveness [7]. However, mechanisms of IgG F0 induction remain unexplored. The roles of bisection, galactosylation and sialylation in tumor-reduction appear less dominant but also remain underexplored.

Here, we investigated various vaccine-adjuvants for tumor-protection and their correlating IgG responses in mice. Tumor-development was induced by i.v. injection of B16-mOVA-cells expressing the transmembrane-bound foreign model protein, ovalbumin (Ova), as a "tumor-neoantigen", thereby excluding antigen-induced self-tolerance mechanisms (Fig. 1A) [8]. For prophylactic vaccination, the soluble Ova protein was injected 12 days prior to tumor-cell injection, together with various adjuvants. 21 days after tumor-cell injection, the number of lung metastases was assessed. Alum and Montanide showed no tumor-protection; MPLA and IFA tended to protect; and Poly(I:C), Alum-Poly(I:C), CFA, and eCFA significantly protected against tumor-growth (Fig. 1B–C). We obtained comparable results when Ova with some adjuvants were administered therapeutically after tumor-cell application (Fig. S1). Additionally, serum transfer from Ova-Poly(I:C)- and Ova-eCFA-immunized mice reduced tumor-growth in other mice (Fig. 1D, E), indicating Ab involvement.

To correlate adjuvant-specific tumor-protection with Ab responses, we analyzed in more detail the Ab responses from a prior experiment [9], in which mice were equally immunized with Ova plus various adjuvants but boosted with Ova alone on day 28 instead of Ova-bearing tumor-cells (Figs. 1F-J, S2, S3, Tabs. S1, S2). Alum and Montanide induced (Th2-driven) IgG1 responses. MPLA, and especially Poly(I:C), induced (Th1) IgG2(c) responses with high IgG2(c)/IgG1 ratios. IFA, and especially eCFA, induced robust IgG1 and IgG2 responses, with Th17-dependent low Fc-galactosylation and -sialylation upon day 14 [9].

Interestingly, all adjuvants induced a transient peak of anti-Ova-IgG1 F0 (up to 5%) after immunization. Furthermore, Poly(I:C) and eCFA induced a second transient peak after boosting. Powerful anti-Ova IgG2(c/b) F0 was not detected. Notably, a transient anti-Spike human IgG1 F0 peak (up to 20%) was observed after the initial SARS-CoV-2 mRNA vaccination, though not upon boosting [10]. To our knowledge, these are the first studies describing that vaccine-adjuvants differentially affect IgG F0 levels after antigen re-exposure.

The adjuvant-specific anti-Ova-IgG subclass levels, ratios and glycosylation patterns of the immunization-experiment on day 14 or 33 (corresponding to the time of “tumor-cell injection” or “antigen re-exposure” in the tumor-experiment, respectively) were then correlated with the number of metastases shown in Fig. 1C (Figs. 1K–M, S4-S7).

**Fig. 2:**
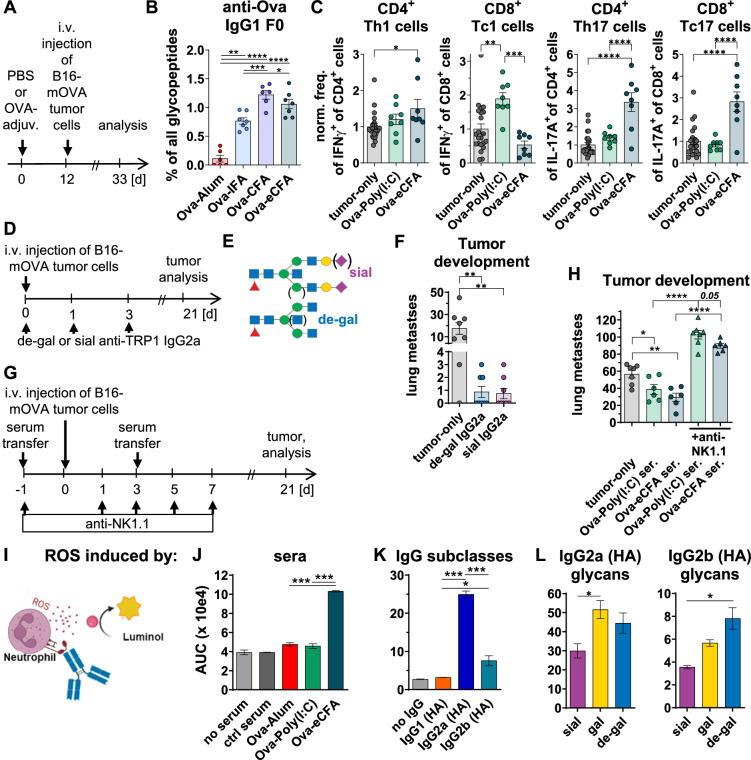
Effects of vaccination and antibodies on tumor-growth, anti-Ova IgG1 F0 and T-cell responses, NK-cell dependency and neutrophil activation. **A**–**C** Anti-Ova IgG1 F0 and T-cell responses after vaccine-adjuvant-induced protection against tumor-growth. (**A**) Experimental design as in Fig. 1A: C57BL/6 mice were immunized i.p. with Ova plus variuos adjuvants. 12 days later, B16-mOVA melanoma cells were injected i.v. and, 21 days after tumor cell injection (day 33), serum anti-Ova IgG1 F0 and splenic T-cell responses were analyzed by nLC-MS or flow cytometry, respectively. (**B**) The percentage of anti-Ova IgG1 afucosylated (F0) glycopeptides of all summed IgG1 glycopeptides analyzed in more detail in one experiment out of the five combined experiments shown in Fig. 1C. No anti-Ova-IgG2 and -IgG3 F0 were detected. (**C**) Normalized (norm.) frequencies (freq.) of the indicated T-cell subsets from two of the five combined experiments shown in Fig. 1C, analyzed in more detail. T-cell subset frequencies were normalized to the respective mean (which was set to 1) of the untreated tumor-group (tumor-only, grey dots) for each experiment. **D**–**F** Treatment of B16-mOVA-bearing mice with glycoengineered anti-TRP1 IgG2a mAbs. (**D**) Experimental design: mice were injected with B16-mOVA-cells and treated with glycoengineered de-galactosylated (de-gal) or galactosylated plus sialylated (sial) anti-TRP1 IgG2a mAbs on days 0, 1 and 3. The number of lung metastases was determined on day 21. (**E**) Schematic of the glycosylation patterns (de-gal and sial) targeted by the glycoengineering of anti-TRP1 IgG2a mAbs. (**F**) Number of metastases. **G**–**H** NK-cell-depletion during serum transfer from Ova-adjuvant-immunized mice to other B16-mOVA-injected mice. (**G**) Experimental design: serum (ser.) was collected from mice 12 days after immunization with Ova-Poly(I:C) or -eCFA and transferred to other mice on days -1 and 3 before/after their B16-mOVA-cell injection. NK-cells were depleted on days − 1, 1, 3, 5 and 7. The number of lung surface metastases was determined on day 21. (**H)** Number of metastases. **I**–**L** Release of reactive oxygen species (ROS) by neutrophils induced by (**J**) sera, (**K**) IgG subclasses (clones HA), and (**L**) glycoengineered (sial, gal and de-gal) IgG2a and IgG2b mAbs in vitro. Statistics in (**B**, **C**, **F**, **J**–**L**): One-way ANOVA with Tukey's multiple comparisons test; statistics in (**H**): unpaired t-tests. *p < 0.05, **p < 0.01, ***p < 0.001, ****p < 0.0001

Across all adjuvants, the anti-Ova-IgG2(c) levels and anti-Ova-IgG1 F0 levels correlated best with tumor-protection. Interestingly, at the end of the tumor-experiment, anti-Ova-IgG1 F0 levels were found to be still higher with eCFA than with Alum (Figs. 2A, B, S8), assuming continuous antigen-re-exposures after B16-mOVA tumor-cell injection.

The data suggest that the antigen-adjuvant immunizations induced a transient extrafollicular IgG1 F0 Ab response but no IgG2 F0 Ab response, and that Poly(I:C) and eCFA induced an IgG1 F0 memory response that can be re-activated after antigen re-exposure(s).

The extent to which murine IgG1 F0, in general, and the low levels of anti-Ova-IgG1 F0 induced in this study, contribute to tumor-protection remains to be elucidated. Due to generally higher F0 levels in humans, suitable vaccine-adjuvants may generate more pronounced variations in human IgG F0 levels and in particular in powerful human IgG1 F0 as mentioned above. Suitable vaccine-adjuvants containing membrane-bound antigens rather than soluble antigens may further increase human IgG1 F0 levels [11].

Tumor-protection induced by IFA and especially eCFA was additionally associated with high levels of all anti-Ova-IgG subclasses with low galactosylation and sialylation, whereas tumor-protection induced by MPLA and especially Poly(I:C) was additionally associated with high IgG2(c)/IgG1 ratios and higher galactosylation and sialylation (Figs. 1L, M, S4-S7). In addition to potentially common protective effects of IgG2(c) and IgG1 F0, these adjuvant-specific characteristics may activate distinct mechanisms.

Analogously, divergent T-cell responses were observed at the tumor-experiment's endpoint shown in Fig. 1C (Figs. 2A, C, S9). Poly(I:C) increased CD8 + IFNγ-expressing Tc1-cell frequencies, whereas eCFA increased Th17- [9] and Tc17-cell frequencies. Both appear to be (bio)markers for low galactosylated IgGs, and vice versa. Notably, Th17-inducing adjuvants are used in autoimmune mouse models to break self-tolerance [12]. They may also be more effective at breaking self-tolerance mechanisms against weak tumor neoantigens, albeit with an increased risk of autoimmune effects.

A comparison of de-galactosylated and galactosylated plus sialylated glyco-engineered anti-TRP1 IgG2a mAbs targeting B16-cells [2], showed strong protection with both glycoforms (Figs. 2D-F, S10), suggesting protection by shared and possibly distinct mechanisms.

NK-cell-depletion not only abolished tumor-protection with serum from Ova-Poly(I:C)- and -eCFA-immunized mice, but even increased tumor-development above control levels, suggesting both vaccination-dependent and -independent anti-tumor-Ab effects by NK-cells (Figs. 2G-H, S11). However, NK-cell-depletion induced more metastases with Poly(I:C)- than eCFA-serum, suggesting a greater involvement of NK-cells in Poly(I:C)-mediated anti-tumor-Ab responses, e.g. via higher IgG2(c)/IgG1 ratios and/or higher levels of galactosylation and sialylation. Consistently, human IgG1-mediated NK-cell-activation is reduced by IgG4 [13] and supposedly enhanced by di-galactosylation [14], as is C1q activation [12]. Conversely, Th17-induced low galactosylated IgGs [9], as observed in inflammatory autoimmunity [12], may promote activation of neutrophils [15], cells which also contribute to tumor-protection in mice and humans [16, 17]. Accordingly, serum from mice immunized with Ova-eCFA, as well as IgG subclass asialylation and, even more strongly, the de-galactosylation of IgG2b, promoted neutrophil activation (Figs. 2I–L, S12, S13).

In conclusion, across all tested adjuvants, tumor protection correlated with the induction of activating IgG2 subclasses and afucosylated IgG1. While all adjuvants initially induced a transient afucosylated IgG1 response, Poly(I:C) and eCFA also induced a second afucosylated IgG1 response upon antigen re-exposure. This response may persist with continuous re-exposures to antigen-expressing tumor-cells. In addition to common protective mechanisms, suitable adjuvants may also induce distinct protective mechanisms, e.g. via NK cells by high IgG2/IgG1 subclass ratios, or di-galactosylated IgGs, or via neutrophils by agalactosylated IgGs.

## Supplementary Information

Supplementary file 1.

## Data Availability

No large datasets were generated or analysed during the current study.

## Abbreviations

Ab: Antibody
Alum: Aluminum hydroxide
CFA: Complete Freund’s adjuvant
eCFA: *M.tb*.-enriched CFA
F0: Afucosylated
GlcNAc: *N*-Acetylglucosamine
IFA: Incomplete Freund’s adjuvant
mAb: Monoclonal antibody
MPLA: Monophosphoryl lipid A
nLC-MS: Nano liquid chromatography-mass spectrometry
Ova: Ovalbumin
PCA: Principle component analysis
Poly(I:C): Polyinosinic-polycytidylic acid
