# Supplementary material for: Anti-tumor vaccine efficacy depends on adjuvant type and associates with induced IgG subclass and glycosylation profiles
Source: Exp Hematol Oncol. 2025 Oct 6;14:122. doi: 10.1186/s40164-025-00708-6 (PMC12502179; doi:10.1186/s40164-025-00708-6)
Supplement: Supplementary file 1 [file 40164_2025_708_MOESM1_ESM.pdf]

Supplementary Information

Anti-tumor vaccine efficacy depends on adjuvant type and associates with induced IgG subclass and glycosylation profiles

Selina Lehrian[a,#], Anna Wasynczuk[b,#], Janina Petry[a], Melanie Guderian[c], Jan Nouta[b], Jana Sophia Buhre[a], Hanna B. Lunding[a], Philipp Köcher[a], Hannah Franziska Schumacher[a], Lara Dühring[a], Kathleen Kurwahn[d], Kristina Manzhula[a], Rudolf Manz[d], Yannic C. Bartsch[c,+], Manfred Wuhrer[b,+], Marc Ehlers[a,e,+]

Material and Methods

Mice

C57BL/6 wild-type (WT) mice were purchased from The Jackson Laboratory and bred in our mouse housing facilities. In addition, C57BL/6 WT mice were purchased from Janvier Labs (Le Genest-Saint-Isle, France). Sex-matched 8- to 12-week-old mice were used in the same experiment.

Ethical considerations

Mouse experiments were performed in accordance with the German animal protection laws and approved by the animal research ethics committees of the respective ministries in Germany.

Reagents

Ovalbumin (Ova) (grade VI; # A2512), 2,4,6-trinitrophenyl (TNP)-Ova, incomplete Freund's adjuvant (IFA) (# F5506), and complete Freund's adjuvant (CFA) (# F5881 (1 mg *Mycobacterium tuberculosis* [*M.tb.*]/mL)) were purchased from Sigma-Aldrich (St. Louis, MO, USA). *M.tb.*-enriched CFA (eCFA) was prepared by adding heat-killed *Mtb.H37RA* (BD Biosciences, San Diego, CA, USA) to IFA (5 mg of *Mtb*/mL). Aluminum hydroxide (Alum) (alhydrogel adjuvant 2%; catalog identifier vac-alu), monophosphoryl lipid A (MPLA) (MPLA-SM VacciGrade; # vac-mpla), and polyinosinic-polycytidylic acid (Poly(I:C)) (HMW VacciGrade; # vac-pic) were purchased from InvivoGen (Toulouse, France). Montanide (# Montanide ISA 51 VG) was purchased from Seppic (Paris, France). Anti-NK1.1 monoclonal antibody (mAb; clone PK136) was purchased from BioXcell (# BE0036; Mannheim, Germany).

Vaccination with Ovalbumin (Ova) plus adjuvant

C57BL/6 mice were immunized intraperitoneally (i.p.) with 100 µg Ova protein plus various adjuvants (Alum, the TLR activators Poly(I:C) and MPLA, the mixture Alum-Poly(I:C), and the more inflammatory water-in-oil adjuvants Montanide, IFA, CFA, and *M.tb.*-enriched (e)CFA) in a total volume of 200 µL, i.e., 100 µL of Ova-PBS solution (1 mg/mL) and 100 µL of the adjuvant solution (e.g., Alum, Montanide, IFA, CFA, eCFA, Poly(I:C) [100 µg in PBS], or MPLA [10 µg in PBS]) were mixed before immunization. Alum-Poly(I:C) was prepared by mixing Alum and the Poly(I:C)-PBS solution 1:1. When indicated, Ova i.p. boosting was performed with 100 µg Ova in 200 µL PBS without adjuvant on day 28. Splenocytes and serum were collected on the indicated days. Serum was frozen at -20°C and splenocytes were kept on ice and analyzed by flow cytometry on the same day.

Tumor cell culture and injection

The C57BL/6 melanoma cell line B16-mOVA stably expressing membrane-bound Ova (**1**) was cultured in DMEM medium (Thermo Fisher (Waltham, MA, USA)) supplemented with 10% fetal bovine serum, 1% penicillin/streptomycin, 4 mM L-glutamine, and 50 µM b-mercaptoethanol at 37°C in 5% CO2. To maintain Ova expression 1.5 mg/mL geneticin was added as a selection marker and cell aliquots were frozen at -80°C. For each experiment, a cell aliquot was thawed and replicated in cell culture for approximately 10 days prior to i.v. injection of 5x10e5 cells into the tail vein of WT mice. 21 days after tumor-cell injection, mice were bled and sacrified, and lungs and spleens were harvested. The number of tumor-cells injected was determined in pre-tests in accordance with the animal research ethics committee of the Ministry of Schleswig-Holstein, Germany, in order to keep the tumour burden within limits until the end of the experiment (21 days after tumor-cell injection), thus avoiding unnecessary suffering to the mice. Serum was frozen and splenocytes were analyzed by flow cytometry on the same day. Lungs were immersed in Fekete's solution (**2**) for 20-24 hours to count lung surface metastases. When multiple experiments were done, the number of metastases was normalized to the mean (which was set to 1) of metastases in the untreated tumor group (tumor-only) or in the control serum-transferred group in each experiment.

Serum transfer

100 µL of pooled sera from Ova-Alum-, -Poly(I:C)-, or -eCFA-immunized C57BL/6 WT mice collected on day 12 or from non-immunized mice (control serum) or only 100 µL of PBS (tumor-cell only group) were injected i.p. into tumor-cell-injected mice on the indicated days.

Anti-Ova- and anti-TNP-IgG subclass ELISAs

ELISA plates were coated with 10 µg/mL of Ova or TNP-Ova to determine the reactivity of serum anti-Ova- or purified monoclonal anti-TNP-IgG subclass Abs, respectively. After incubation with the indicated serum or mAb dilutions, bound Abs were detected with horseradish peroxidase (HRP)-

coupled polyclonal goat anti-mouse IgG-Fc-specific, IgG1-specific, IgG2c-specific (the isoform of IgG2a in C57BL/6 mice), or IgG2b-specific Abs (all from Bethyl Laboratories, Montgomery, TX, USA). After incubation with 3,3′,5,5′-tetramethylbenzidine (TMB) substrate (BD Biosciences, San Diego, Calif, USA), the OD was measured at 450 nm.

Analysis of IgG-Fc glycosylation via nano liquid chromatography-mass spectrometry (nLC-MS)

IgG-Fc subclass glycopeptides were measured by nano liquid chromatography-mass spectrometry (nLC-MS) for the indicated immunization groups on the indicated days in a previous study and re-analyzed here (**3,4**). Briefly, anti-Ova Abs were affinity-captured from sera using Sepharose-coupled Ova or in-house generated Ova-coated plates (Thermo Fisher Scientific, Roskilde, Denmark). Ab elution was performed using 100 mM formic acid. Eluates from anti-Ova-Ab affinity purification were dried by vacuum centrifugation and subjected to tryptic cleavage followed by nLC-MS. The IgG1 and IgG2 (both IgG2c and IgG2b, which could not be distinguished by the glycopeptide profiling method used) subclass-specific Fc *N*-glycopeptide peak areas were background-corrected and assigned to one of the following 12 glycan compositions: G0F, G0F0, G1F, G1F0, G2, G2F, G3F, G4F, G1S1F, G2S1F, G3S1F, and G2S2F (**Suppl. Table S1**). The summed intensities of the IgG subclass glycopeptides were used to calculate absolute anti-Ova-IgG1 and IgG2 levels and ratios. The relative intensity of each glycopeptide was calculated by normalizing the glycopeptide intensity to the sum intensity of all glycopeptides of the corresponding subclass (total area normalization). The relative intensities of the IgG subclass glycopeptides were used to calculate the glycosylation traits: fucosylation, galactosylation, and sialylation (**Suppl. Table S2**). The afucosylated glycopeptide signals were detected only for anti-Ova-IgG1, not for IgG2. Notably, the suitability of our method for detecting IgG2 F0 was recently demonstrated by the detection of monoclonal murine IgG2 F0 patterns (**5**). The G3F, G4F, and G3S1F glycopeptide signals (carrying additional alpha1,3-linked terminal galactose(s)) were also detected only for anti-Ova-IgG1 with summed relative peak area intensities always less than 3%. Glycopeptides containing bisecting *N*-acethylglucosamine (GlcNAc) were not detected.

In vitro de-sialylation and additional sialylation of murine IgG monoclonal antibodies

Anti-TNP murine IgG1, IgG2a, and IgG2b hybridoma switch variant mAbs (clones HA) with identical V(D)J sequences, an anti-TNP murine IgG1 hybridoma mAb (H5), and anti-erythrocyte murine IgG2a, and IgG2b hybridoma switch variant mAbs (34-3C) with identical V(D)J sequences (**6-8**) were purified from the hybridoma supernatants using Protein G-sepharose (GenScript Corporation, Piscataway, NJ, USA). Anti-TRP1 IgG2a mAb (clone TA99) was purchased (BioXcell).

IgG subclass mAbs were de-sialylated (de-sial) with sialidase A (#GK80040; Agilent, Santa Clara, CA, USA) and additionally de-galactosylated (de-gal) with β(1-4)-galactosidase (#GKX-5014; Agilent), as recently described (**3,7,9,10**). Additional galactosylation (gal) or galactosylation plus sialylation (sial) of purified IgG subclass mAbs were performed in a one- or two-step procedure, respectively, as previously described (**3,7,9,10**). Briefly, purified mAbs were additionally galactosylated (gal) with human β-1,4-galactosyltransferase (Roche, Basel, Switzerland) and UDP-galactose (Merck KGaA, Darmstadt, Germany) and subsequently sialylated (sial) with human α-2,6-sialyltransferase (Roche) and CMP-sialic acid (Merck KGaA). The different mAb glycoforms were purified by size exclusion (using centrifugal filter units; pore size 100 KDa) or Protein G as described above. The mAb glycoforms were verified by SNA-lectin ELISA and anti-TNP ELISA.

Sialylation analysis of murine IgG subclass mAbs by SNA-lectin ELISA

ELISA plates were coated with serial dilutions of IgG subclass mAb glycoforms. Sialylation was detected with HRP-coupled *Sambucus nigra* lectin (SNA; Biomol, Hamburg, Germany) (**10**). After incubation with TMB (BD Biosciences), the optical density (OD) was measured at 450 nm.

Tumor treatment with anti-TRP1 IgG2a monoclonal antibody glycoforms

For tumor treatment with anti-TRP1 IgG2a mAb (clone TA99; which binds to an intrinsic tumor-cell antigen of B16-cells), mice were injected i.p. with 100 µg of glycoengineered mAb forms (de-galactosylated (de-gal) vs galactosylated plus sialylated (sial)) on day 0 and day 1 after tumor-cell (B16-mOva) injection and additionally with 50 µg on day 3 after tumor-cell injection.

NK-cell depletion

NK-cells were depleted in C57BL/6 mice by i.p. injection of 200 µg of anti-NK1.1 mAb (clone PK136) on day -1, prior to tumor cell injection, and 100 µg on days 1, 3 and 5, and 65 µg on day 7, after tumor cell injection. NK-cell depletion was analyzed by flow cytometry on day 6.

Neutrophil ROS activation assay

The murine neutrophil activation assay was performed as recently described (**11**). Briefly, to isolate neutrophils, murine femurs were harvested from naive C57BL/6J mice, and bone marrow cells were rinsed with sterile murine neutrophil buffer (MNB; HBSS (Life Technologies) containing 0.1% BSA (PAN-Biotech) and 1% glucose (Merck Millipore)) and filtered through a 70 µm cell strainer. For density gradient centrifugation, Ficoll (GE Healthcare) with four different densities (1.116, 1.104, 1.098, and 1.094) were stacked to obtain a gradient. Isolated bone marrow cells were washed with MNB, resuspended in Ficoll (p = 1.091) and layered on top of the prepared Ficoll gradient. After centrifugation (1,600 x g, 10°C, 30 min, brake off), the cells between the two lower Ficoll layers were collected and washed twice with MNB. The cells were then resuspended in MNB, loaded onto Histopaque 1119

(Sigma-Aldrich), and centrifuged again as described above. Neutrophils accumulated above the Histopaque 1119 were harvested and washed with MNB.

For the ROS (reactive oxygen species) release assay, a 96-well plate (LUMITRAC Corning 3922, Greiner BioOne) was coated with 10 µg/mL Ovalbumin or 10 µg/mL of the in vitro glycoengineered IgG subclass mAbs for 1 h at RT and washed three times with PBS/0.05% Tween 20 (Sigma-Aldrich). Blocking was performed with PBS/2.5% milk for 1 hour at RT, followed by three washes. Diluted sera as indicated were added to the Ova-coated plate for 1 h at RT, followed by three washes. 200,000 isolated neutrophils in MNB containing 0.2 mg/mL 1x luminol (Sigma-Aldrich) were added to each well and luminescence was measured immediately and over 1.5 h at 37°C in a Spectra Max iD3 ELISA reader (Molecular Devices, LLC. (San Jose, CA, USA)).

Flow cytometric analysis

For T-cell intracellular cytokine analysis, splenocytes were restimulated with a cell stimulation cocktail containing phorbol myristate acetate (PMA) (catalog identifier tlrl-pma, InvivoGen (Toulouse, France)) and ionomycin (catalog no. 73724, StemCell Technologies (Vancouver, Canada)) and protein transport inhibitors (brefeldin A (catalog no. v420601, Biolegend (San Diego, CA, USA)), monensin (catalog no. 420.701, Biolegend) according to the manufacturer's instructions prior to subsequent flow cytometric analysis (Attune Nxt, Thermo Fisher Scientific). The following fluorochrome-coupled Abs were used for surface staining at 4°C: anti-B220 AF488 or BV785 (Biolegend, clone RA3-6B2), anti-CD4 BV785 (Biolegend, RM4-5), and anti-CD8α AF700 (BioLegend, 53-6.7). Cells were then fixed and permabilized with Cytofix/Cytoperm™ (catalog no. 554722, BD Bioscience) according to the manufacturer's instructions and stained with anti-IFNγ AF647 (BD Biosciences, XMG1.2) and anti-IL-17A PE (eBioscience, eBio17B7). For T-cells analysis, spleen cells were pre-gated on live and $B220^-$ cells.

For splenic NK-cell or bone marrow neutrophil analysis, cells were stained with anti-NK1.1 AF700 (Biolegend, PK136), anti-Gr-1 BV605 (Biolegend RB6-8C5), anti-CD3 BV785 (Biolegend, 17A2), anti-B220 BV785 (Biolegend, RA3-6B2), anti-CD11b BV421 (Biolegend, M1/70), anti-FcγRIII PE (Biolegend, 275003), and anti-FcγRIV FITC (Biolegend, 9R9).

Principal Component Analysis (PCA)

PCA was used to reduce the large dataset of Abs features and normalized lung metastasis numbers, while retaining significant patterns and trends, to creat a smaller, more manageable dataset. The data were transformed into a new coordinate system showing the greatest variations. The axes of the coordinate system represent "Principal Components" (PC), with PC1 representing the most variation in the data and PC2 representing the second most variation in the data.

Data availability

All data are available in the manuscript. Additional information is available from the corresponding author.

Statistical analysis

Statistical analyses were performed by using GraphPad Prism software, version v10.0 (GraphPad, La Jolla, CA). A normal distribution was assumed for the small sample sizes. The 2-tailed Student t test or one-way ANOVA was used to analyze differences between two normally distributed groups or between more than two groups, respectively. Pearson correlations with a 95% confidence interval were used to assess the linear relationship between two variables. P-values < 0.05 were considered significant as follows: *, **, ***, ****: p-value < 0.05, 0.01, 0.001, and 0.0001, respectively. Unless otherwise noted, mouse data were taken from one representative experiment of 2-5 individual experiments or combined from multiple experiments and presented as mean +/-SEM, as indicated.

Abbreviations

Ab: antibody; Alum: aluminum hydroxide; CFA: complete Freund's adjuvant; eCFA: *M.tb*.-enriched CFA; Fc: fragment crystallizable; F0: afucosylated; GlcNAc: *N*-acethylglucosamine; IFA: incomplete Freund's adjuvant; IgG: immunoglobulin G; mAb: monoclonal antibody; MPLA: monophosphoryl lipid A; *M.tb.: Mycobacterium tuberculosis*; nLC-MS: nano liquid chromatography-mass spectrometry; Ova: ovalbumin; PCA: principal component analysis; Poly(I:C): polyinosinic-polycytidylic acid; ROS: reactive oxygen species; SNA: *Sambucus nigra* lectin.

Supplementary References

1. DiLillo DJ, Yanaba K, Tedder TF. B Cells Are Required for Optimal CD4+ and CD8+ T Cell Tumor Immunity: Therapeutic B Cell Depletion Enhances B16 Melanoma Growth in Mice. The Journal of Immunology 2010; 184(7):4006–16. doi: 10.4049/jimmunol.0903009. PMID: 20194720.
2. Overwijk WW, Restifo NP. B16 as a Mouse Model for Human Melanoma. Current Protocols in Immunology 2000; 39(1):20.1.1-20.1.29. doi: 10.1002/0471142735.im2001s39. PMID: 18432774.
3. Bartsch YC, Eschweiler S, Leliavski A, Lunding HB, Wagt S, Petry J, Lilienthal GM, Rahmöller J, de Haan N, Hölscher A, Erapaneedi R, Giannou AD, Aly L, Sato R, de Neef LA, Winkler A, Braumann D, Hobusch J, Kuhnigk K, Krémer V, Steinhaus M, Blanchard V, Gemoll T, Habermann JK, Collin M, Salinas G, Manz RA, Fukuyama H, Korn T, Waisman A, Yogev N, Huber S, Rabe B, Rose-John S, Busch H, Berberich-Siebelt F, Hölscher C, Wuhrer M, Ehlers M. IgG Fc sialylation is regulated during the germinal center reaction following immunization with different adjuvants. J

Allergy Clin Immunol 2020; 146(3):652-666.e11. doi: 10.1016/j.jaci.2020.04.059. PMID: 32445838.

4. Falck D, Wuhrer M. GlYcoLISA: antigen-specific and subclass-specific IgG Fc glycosylation analysis based on an immunosorbent assay with an LC-MS readout. Nat Protoc. 2024; 19(6):1887-1909. doi: 10.1038/s41596-024-00963-7. PMID: 38383719. Review.
5. Dekkers G, Bentlage AEH, Plomp R, Visser R, Koeleman CAM, Beentjes A, Mok JY, van Esch WJE, Wuhrer M, Rispens T, Vidarsson G. Conserved FcγR- glycan discriminates between fucosylated and afucosylated IgG in humans and mice. Mol Immunol. 2018; 94:54-60. doi: 10.1016/j.molimm.2017.12.006. PMID: 29268168.
6. Strait RT, Posgai MT, Mahler A, Barasa N, Jacob CO, Köhl J, Ehlers M, Stringer K, Shanmukappa SK, Witte D, Hossain MM, Khodoun M, Herr AB, Finkelman FD. IgG1 protects against renal disease in a mouse model of cryoglobulinemia. Nature 2015; 517(7535):501-504. doi: 10.1038/nature13868. PMID: 25363774. Erratum: Nature 2015; 526 (7575): 728. doi: 10.1038/nature15534. PMID: 26416751.
7. Epp A, Hobusch J, Bartsch YC, Petry J, Lilienthal G-M, Koeleman CAM, Eschweiler S, Möbs C, Hall A, Morris SC, Petzold D, Engellenner C, Bitterling J, Rahmöller J, Leliavski A, Thurmann R, Collin M, Moremen KW, Strait RT, Blanchard V, Petersen A, Gemoll T, Habermann JK, Petersen F, Nandy A, Kahlert H, Hertl M, Wuhrer M, Pfützner W, Jappe U, Finkelman FD, Ehlers M. Sialylation of IgG antibodies inhibits IgG-mediated allergic reactions. J Allergy Clin Immunol 2018; 141(1): 399-402.e8. doi: 10.1016/j.jaci.2017.06.021. PMID: 28728998.
8. Azeredo da Silveira S, Kikuchi S, Fossati-Jimack L, Moll T, Saito T, Verbeek JS, Botto M, Walport MJ, Carroll M, Izui S. Complement activation selectively potentiates the pathogenicity of the IgG2b and IgG3 isotypes of a high affinity anti-erythrocyte autoantibody. J Exp Med. 2002; 195(6):665-72. doi: 10.1084/jem.20012024. PMID: 11901193.
9. Petry J, Rahmöller J, Dühring L, Lilienthal G-M, Lehrian S, Buhre JS, Bartsch YC, Epp A, Lunding HB, Moremen KW, Leliavski A, Ehlers M. Enriched blood IgG sialylation attenuates IgG-mediated and IgG-controlled-IgE-mediated allergic reactions. J Allergy Clin Immunol 2021; 147(2):763–7. doi: 10.1016/j.jaci.2020.05.056. PMID: 32603664.
10. Dühring L, Petry J, Lilienthal G-M, Bartsch YC, Kubiak M, Pfeufer C, Lehrian S, Buhre JS, Lunding HB, Kern C, Behrends J, Walsemann T, Gädert L, Sommer C, Krüger L, Blanchard V, Dehmel S, Jappe U, Rahmöller J, Ehlers M. Sialylation of IgE reduces FcεRIα binding and mast cell activation in vitro and increases IgE half-live in vivo. Allergy 2023; 78(8):2301. doi: 10.1111/all.15665. PMID: 36724158.
11. Clauder A-K, Kordowski A, Bartsch YC, Köhl G, Lilienthal G-M, Almeida LN, Lindemann T, Petry J, Rau CN, Gramalla-Schmitz A, Dühring L, Elbracht C, Kenno S, Tillmann J, Wuhrer M, Ludwig R, Ibrahim SM, Bieber K, Köhl J, Ehlers M, Manz R. IgG Fc N-glycosylation translates

MHCII haplotype into autoimmune skin disease. J Invest Dermatology 2021; 141(2):285-294. doi: 10.1016/j.jid.2020.06.022. PMID: 32653301.

Supplementary Figures and Tables

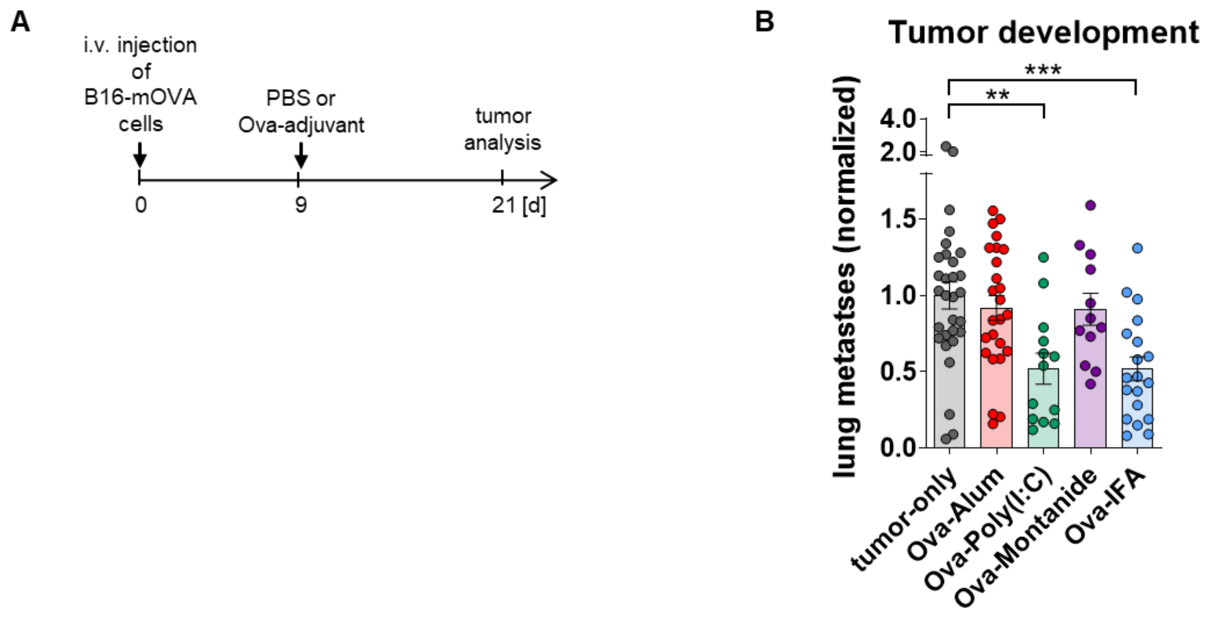

Suppl. Figure S1: Therapeutic vaccine-adjuvant-induced protection against tumor growth. **(A)** Experimental design: C57BL/6 mice were injected i.v. with B16-mOVA melanoma cells on day 0 immunized i.p. with Ova plus different adjuvants on day 9 and analyzed on day 21. **(B)** Normalized number of surface lung metastasis from multiple experiments. Statistics: One-way ANOVA with Dunett's multiple comparisons test. *p < 0.05, **p < 0.01, ***p < 0.001, ****p < 0.0001.

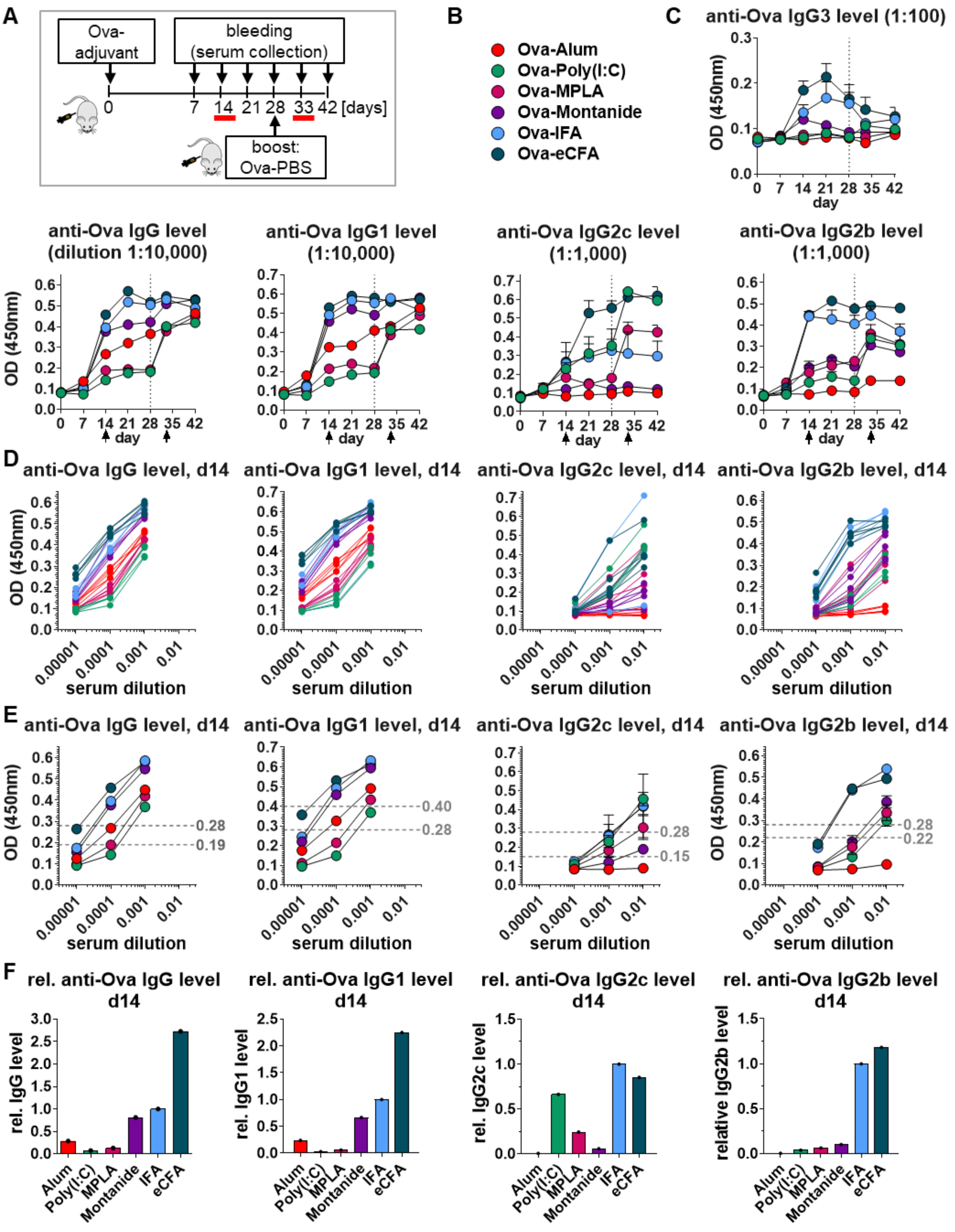
A
Ova-adjuvant
bleeding (serum collection)
0
7 14 21 28 33 42 [days]
boost: Ova-PBS
B
Ova-Alum
Ova-Poly(I:C)
Ova-MPLA
Ova-Montanide
Ova-IFA
Ova-eCFA
C
anti-Ova IgG3 level (1:100)
OD (450nm)
day
anti-Ova IgG level (dilution 1:10,000)
anti-Ova IgG1 level (1:10,000)
anti-Ova IgG2c level (1:1,000)
anti-Ova IgG2b level (1:1,000)
OD (450nm)
day
D
anti-Ova IgG level, d14
anti-Ova IgG1 level, d14
anti-Ova IgG2c level, d14
anti-Ova IgG2b level, d14
OD (450nm)
serum dilution
E
anti-Ova IgG level, d14
anti-Ova IgG1 level, d14
anti-Ova IgG2c level, d14
anti-Ova IgG2b level, d14
OD (450nm)
0.28
0.19
0.40
0.28
0.28
0.15
0.28
0.22
serum dilution
F
rel. anti-Ova IgG level d14
rel. anti-Ova IgG1 level d14
rel. anti-Ova IgG2c level d14
rel. anti-Ova IgG2b level d14
rel. IgG level
rel. IgG1 level
rel. IgG2c level
relative IgG2b level
Alum
Poly(I:C)
MPLA
Montanide
IFA
eCFA

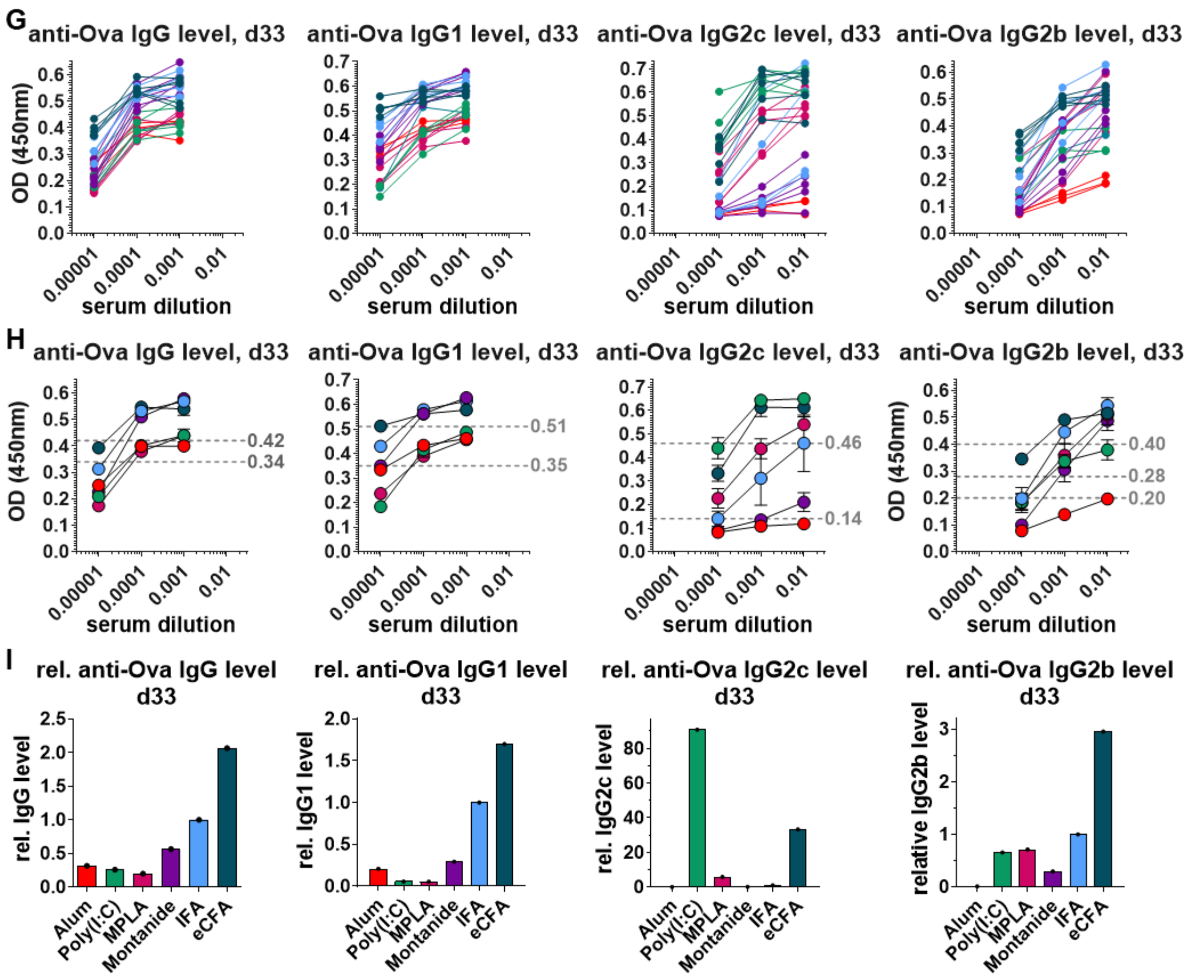

Suppl. Figure S2: Anti-Ova IgG and IgG subclass levels after Ova immunization with different adjuvants over time and calculation of relative levels on day 14 and day 33.

(A) Experimental design: C57BL/6 mice were immunized i.p. with Ova plus different adjuvants, boosted on day 28 with Ova without adjuvant and blood sera were collected on the indicated days (**3**). **(B)** Color coding of the adjuvants studied. **(C)** Sera were analyzed for anti-Ova-IgG and -IgG subclass levels using ELISA absorbance values (OD450nm) at defined serum dilutions. Sera were further analyzed to calculate more precise relative anti-Ova-IgG and -IgG subclass levels on days 14 and 33 post immunization (black arrows) using serum dilution series curves. **(D and G)** Anti-Ova-IgG and -IgG subclass ELISA absorbance values (OD450nm) of each sample on (D) day 14 and (G) day 33 post-immunization were analyzed as serum dilution series curves. **(E and H)** The corresponding mean values of each adjuvant group on (E) day 14 and (H) day 33 were calculated for each serum dilution. To compare the Ab levels of the different adjuvant groups, relative (rel.) anti-Ova-IgG and -IgG subclass levels were calculated by determining the x-axis intersection value of each adjuvant mean curve at two defined y-axis intersections. The x-axis intersection value of each y-axis intersection was normalized to the calculated x-axis intersection (set to 1) of Ova-IFA. **(F and I)** The reciprocal values are shown as relative anti-Ova-IgG and -IgG subclass levels of each adjuvant group on (F) day 14 and (I) day 33.

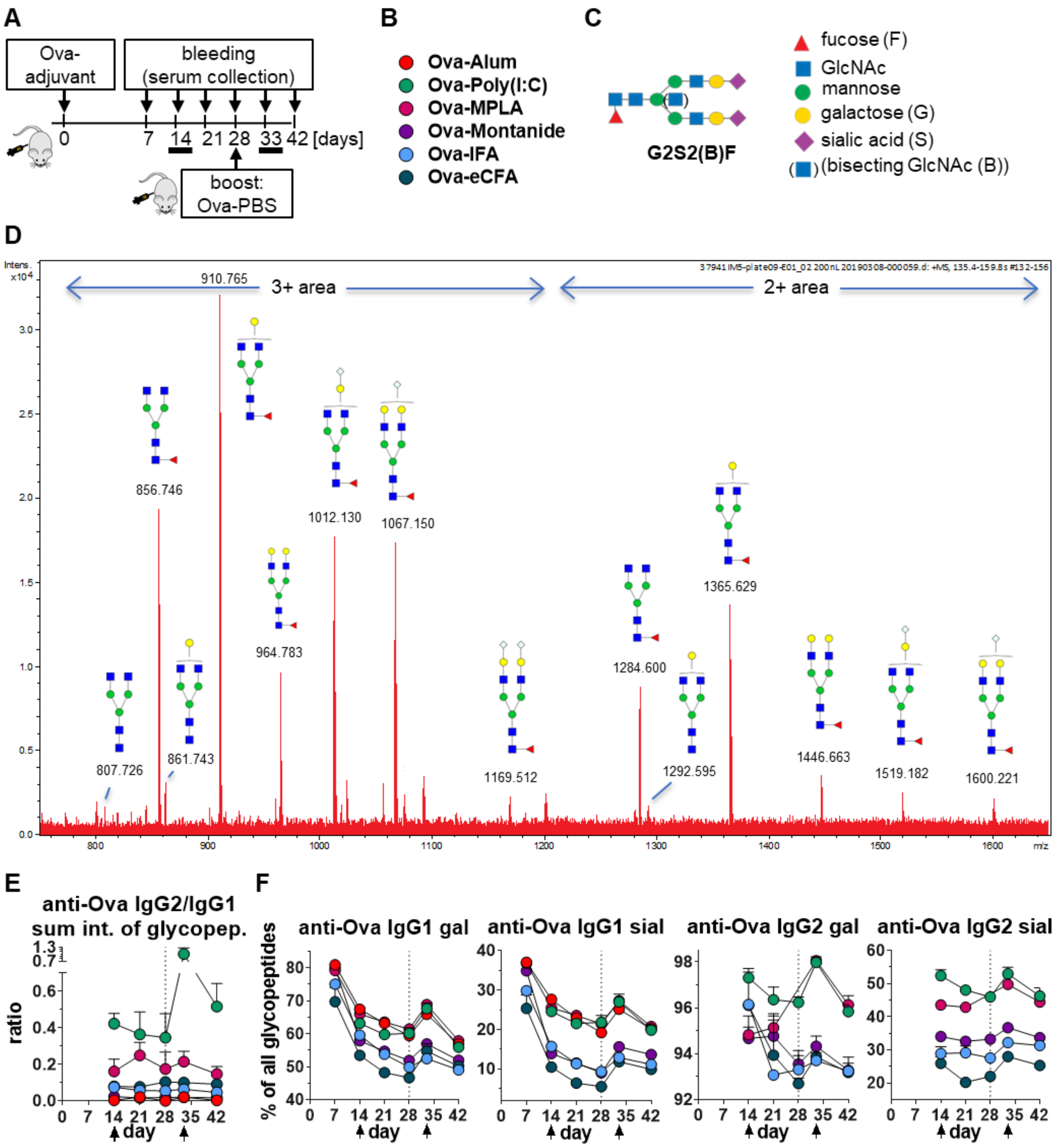

Suppl. Figure S3: IgG subclass ratios and Fc glycosylation patterns after Ova immunization with different adjuvants.

(A) Experimental design: C57BL/6 mice were immunized i.p. with Ova plus different adjuvants, boosted on day 28 with Ova without adjuvant and blood sera were collected on the indicated days (n=5 per group) (**3**). **(B)** Color code of the adjuvants studied. **(C)** Schematic representation of the glycosylation pattern G2S2(B)F. **(D)** IgG1 glyocopeptide peaks (3+ and 2+) of an Ova-Alum sample on day 7, as measured by nLC-MS. **(E)** Ratio of anti-Ova-IgG2 to -IgG1 sum intensity levels shown in Figure 1H. **(F)** Anti-Ova-IgG1 and -IgG2 galactosylation and sialylation percentages of all corresponding subclass glycopeptides over time (**3**).

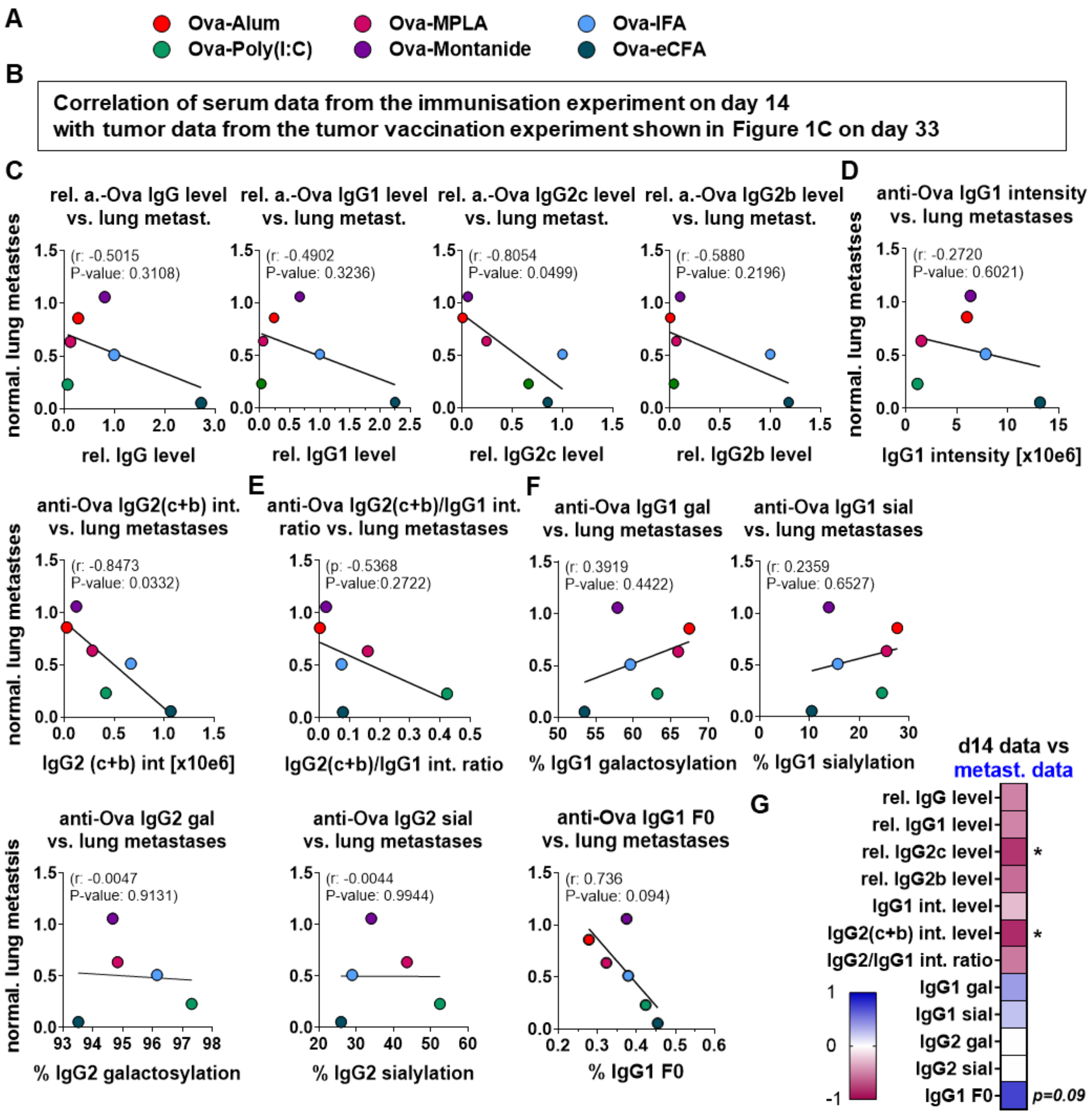

Suppl. Figure S4: Correlation of anti-Ova-IgG and -IgG subclass (relative) levels, sum intensity levels, sum intensity ratios and glycosylation patterns of the immunization experiment on day 14 and relative numbers of lung surface metastases of the tumor vaccination experiment on day 33. **(A)** Color code for the adjuvants studied. **(B)** Note which data were correlated. **(C-F)** Means of each adjuvant group of the anti-Ova-IgG and -IgG subclass (C) relative levels, (D) sum intensity levels, (E) sum intensity ratios and (F) glycosylation pattern percentages from the immunization experiment in **Figure 1F-I** and **Suppl. Figures S2 and S3** on day 14 were correlated with the adjuvant-specific means of the normalized (norm.) numbers of lung surface metastases of the tumor experiment as shown in **Figure 1C**. Pearson's correlation coefficients "r" between -1 and 1 and p-values are shown. **(G)** Heat map of Pearson's correlations shown in (C-F). Selected p-values are shown. Statistics: Pearson's correlation. *$p < 0.05$.

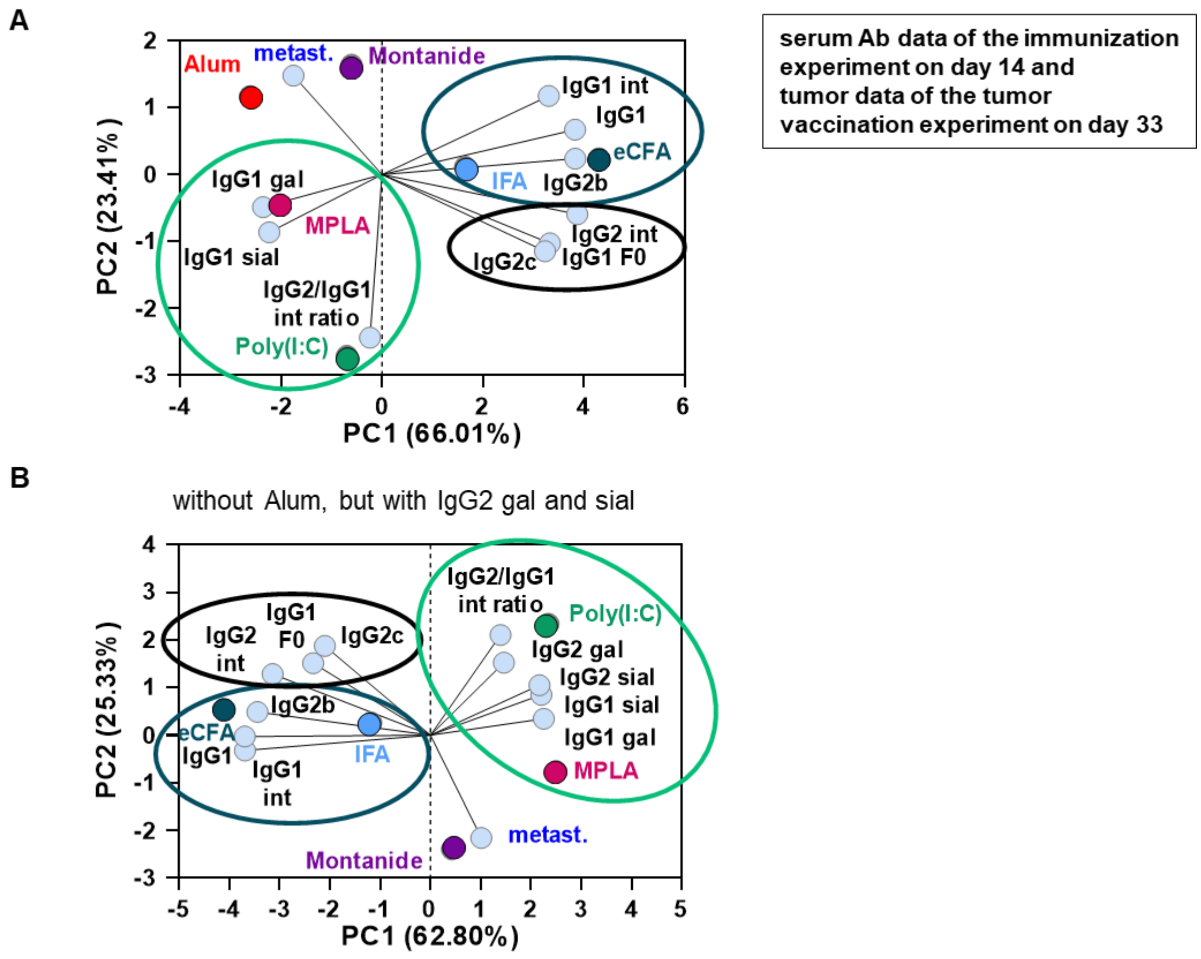

Suppl. Figure S5: PCA analysis of the Ab data sets from the immunization experiment on day 14 and the normalized number of lung metastases from the tumor experiment on day 33.
(A) PCA of the Ab datasets from the immunization experiment on day 14 shown in Figure 1F-I and Suppl. Figures S2 and S3 and the normalized number of lung metastases of the tumor experiment shown in Figure 1C (loadings) showing each loading and the score for each adjuvant. **(B)** PCA as in (A) but without the Alum dataset allowing additional analysis of the anti-Ova-IgG2 gal and sial data.

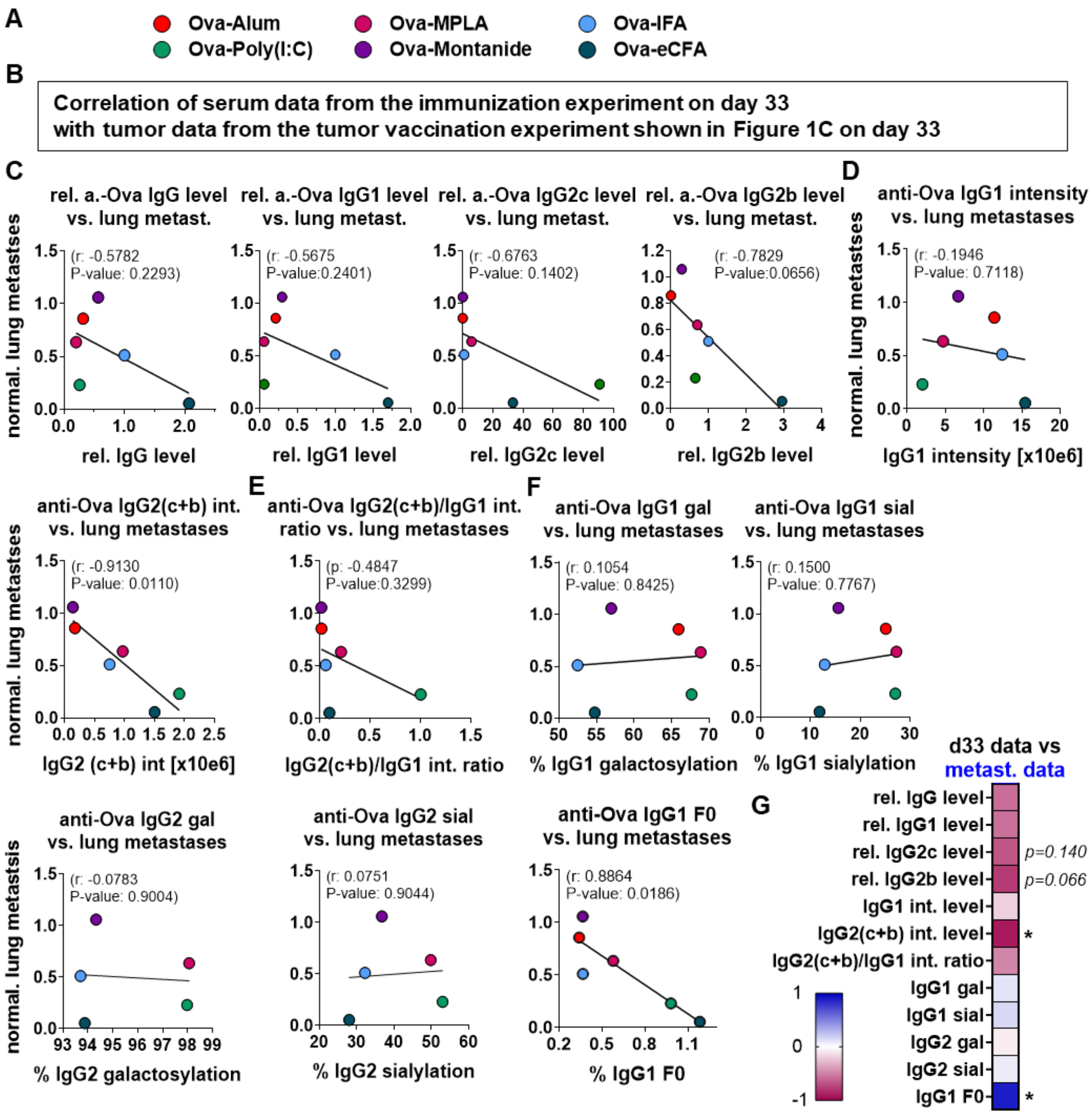

Suppl. Figure S6: Correlation of anti-Ova-IgG and -IgG subclass (relative) levels, sum intensity levels, sum intensity ratios and glycosylation patterns of the immunization experiment on day 33 and relative numbers of lung surface metastases of the tumor vaccination experiment on day 33. **(A)** Color code for the adjuvants studied. **(B)** Note which data were correlated. **(C-F)** Means of each adjuvant group of the anti-Ova-IgG and -IgG subclass (C) relative levels, (D) sum intensity levels, (E) sum intensity ratios and (F) glycosylation pattern percentages from the immunization experiment in Figure 1F-I and Suppl. Figures S2 and S3 on day 33 were correlated with the adjuvant-specific means of the normalized (norm.) numbers of lung surface metastases of the tumor experiment as shown in Figure 1C. Pearson's correlation coefficients "r" between -1 and 1 and p-values are shown. **(G)** Heat map of Pearson's correlations shown in (C-F). Selected p-values are shown. Statistics: Pearson's correlation. *$p < 0.05$.

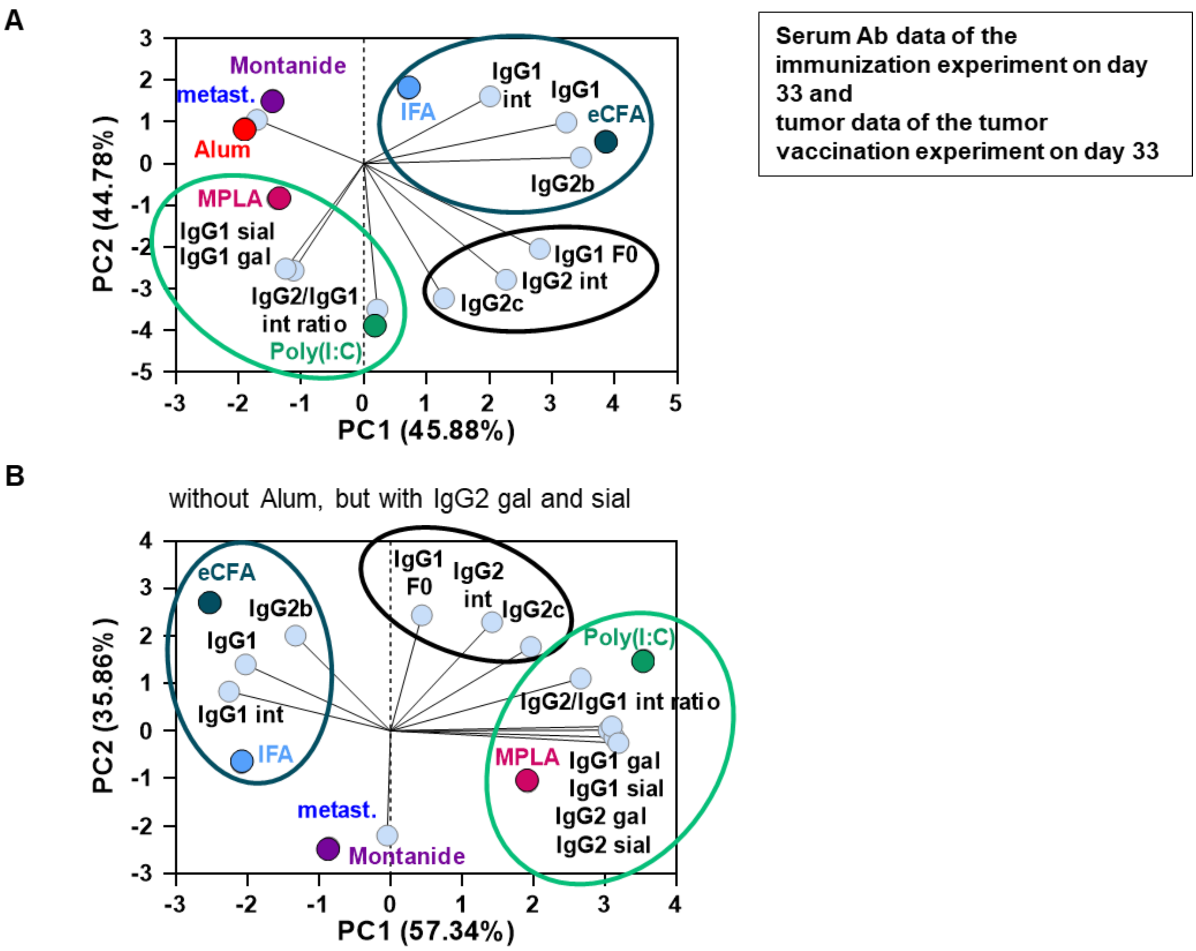

Suppl. Figure S7: PCA analysis of the Ab data sets from the immunization experiment on day 33 and the normalized number of lung metastases from the tumor experiment on day 33.
(A) PCA of day 33 Ab data sets from the immunization experiment shown in Figure 1F-I and Suppl. Figures S2 and S3 and the normalized number of lung metastases of the tumor experiment shown in Figure 1C (loadings) showing each loading and the score for each adjuvant. **(B)** PCA as in (A) but without the Alum dataset allowing additional analysis of the anti-Ova-IgG2 gal and sial data.

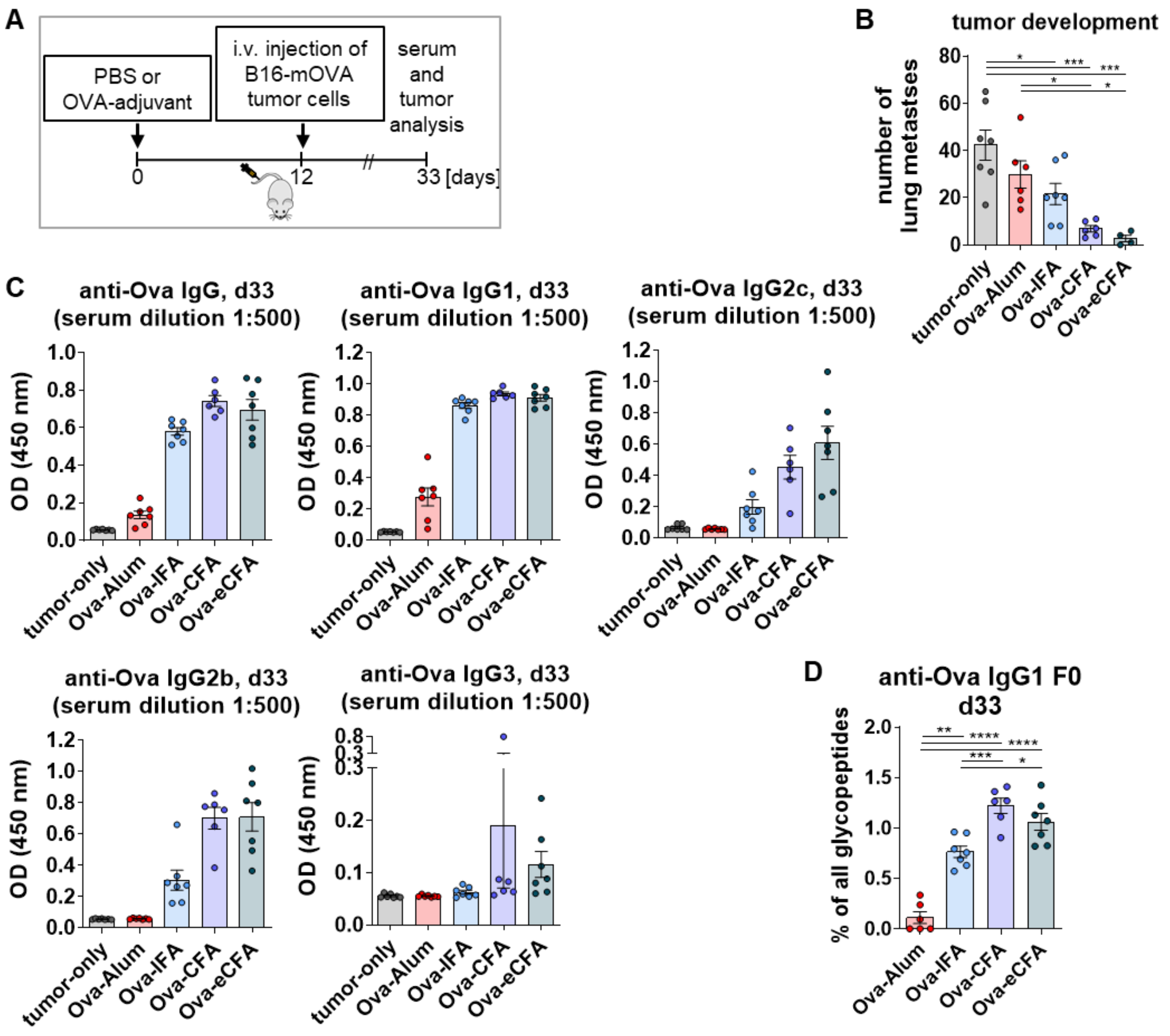

Suppl. Figure S8: Vaccine-adjuvant-induced protection against tumor growth and IgG antibody responses.

(A) Experimental design: C57BL/6 mice were immunized i.p. with Ova plus different adjuvants on day 0, injected i.v. with B16-mOVA melanoma cells and analyzed on day 33. The following data shown belong to one of the five combined experiments shown in Figure 1C. **(B)** Number of lung metastases. **(C)** Anti-Ova IgG and IgG subclass ELISA with the indicated serum dilutions. **(D)** Percentage of anti-Ova IgG1 afucosylated (F0) glycopeptides of all summed IgG1 glycopeptides (also shown in Figure 2B). No anti-Ova-IgG2 and -IgG3 F0 was detected. Statistics: One-way ANOVA with Tukey's multiple comparisons test. *p < 0.05, **p < 0.01, ***p < 0.001, ****p < 0.0001.

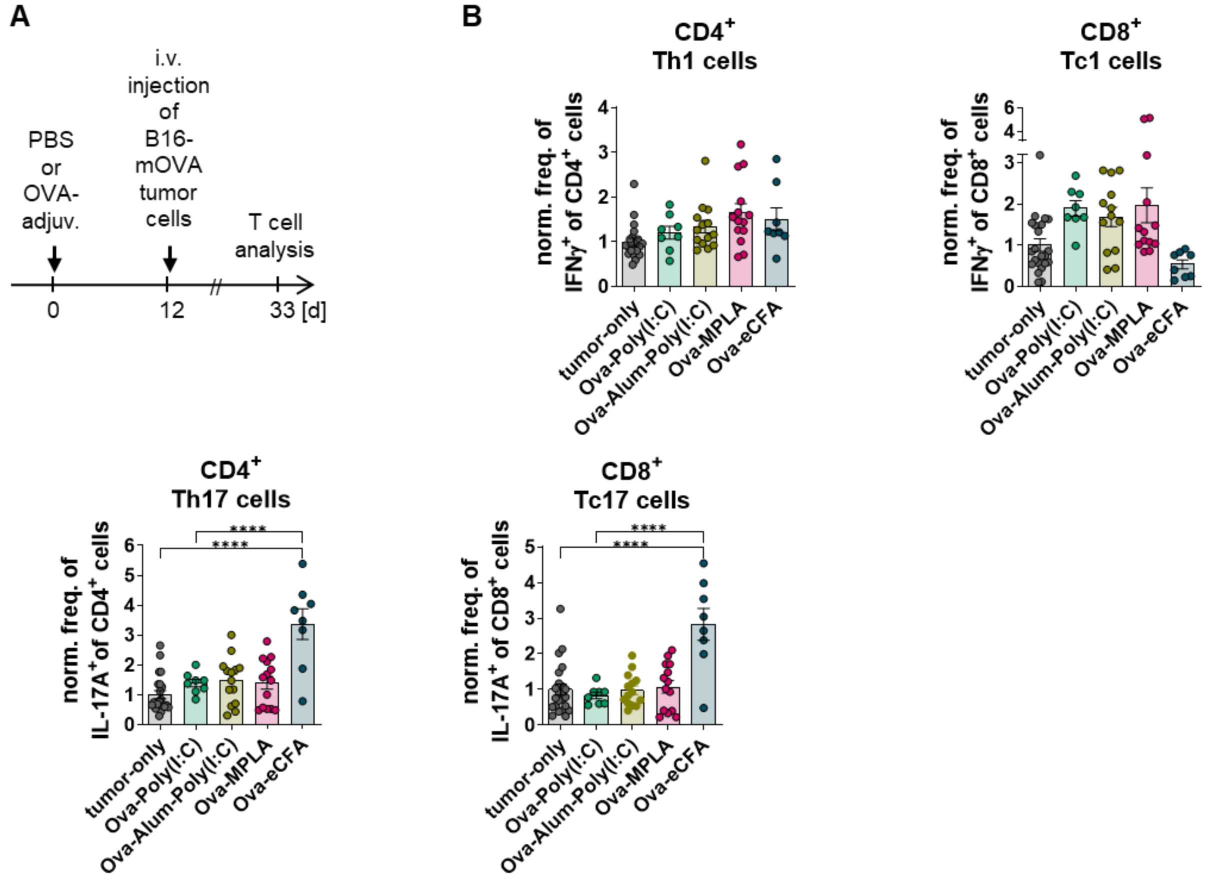

Suppl. Figure S9: T cell responses following vaccine-adjuvant-induced protection against tumor-growth. (A) The experimental design is the same as in Figures 1A and Figure 2A. C57BL/6 mice were immunized i.p. with Ova plus various adjuvants. 12 days later, B16-mOVA melanoma cells were injected i.v. and, 21 days after tumor cell injection (day 33), splenic T cell responses were analyzed by flow cytometry. **(B)** Normalized (norm.) frequencies (freq.) of the indicated T cell subsets from two of the five combined experiments shown in Figure 1C analyzed in more detail. Neither of the two experiments contained the Ova-Alum, Ova-Montanide, Ova-IFA or Ova-CFA groups. T cell subset frequencies were normalized to the respective mean (set to 1) of the untreated tumor-group (tumor-only, grey dots) for each experiment. Statistics: One-way ANOVA with Tukey's multiple comparison test: *p < 0.05, **p < 0.01, ***p < 0.001, ****p < 0.0001.

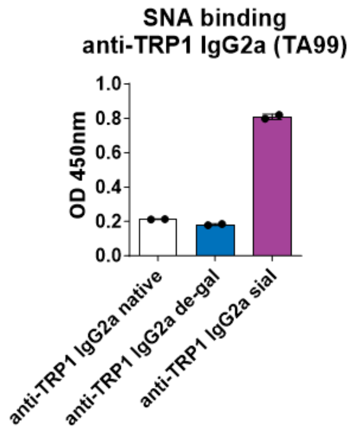

Suppl. Figure S10: Characterization of the differentially glycoengineered anti-TRP1 IgG2a monoclonal antibodies used.

Analysis of differentially glycoengineered (native, de-gal and sial) anti-TRP1 IgG2a mAbs for sialylation by SNA ELISA (SNA binds to sialic acid), where mAbs were coated directly onto the plate (1 µg/ml) and sialylation was detected by SNA-HRP. Each sample was run in duplicate.

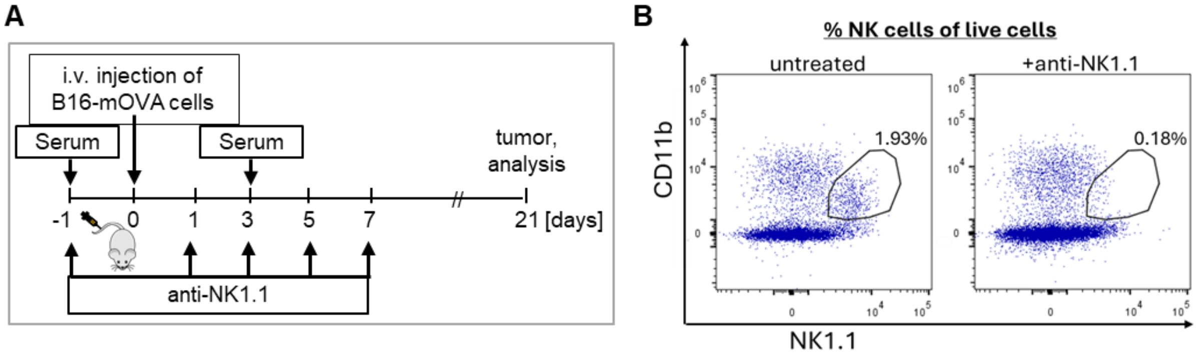

Suppl. Figure S11: Verification of NK-cell depletion from the experiment shown in Fig. 6.

(A) Experimental design: Serum was collected from C57BL/6 mice 12 days after immunization with Ova-Poly(I:C) or -eCFA. Sera were transferred to other C57BL/6 mice on day -1 before and 3 days after B16-mOVA cell injection. For NK-cell depletion, mice were additionally injected with 200 µg anti-NK1.1 Abs on day -1 and 100 µg on days 1, 3, 5 and 7. On day 6, splenocytes (pre-gated on live cells) were analyzed for NK-cell ($CD11b^{+}$ $NK1.1^{+}$) depletion by flow cytometry. **(B)** Representative NK-cell stainings.

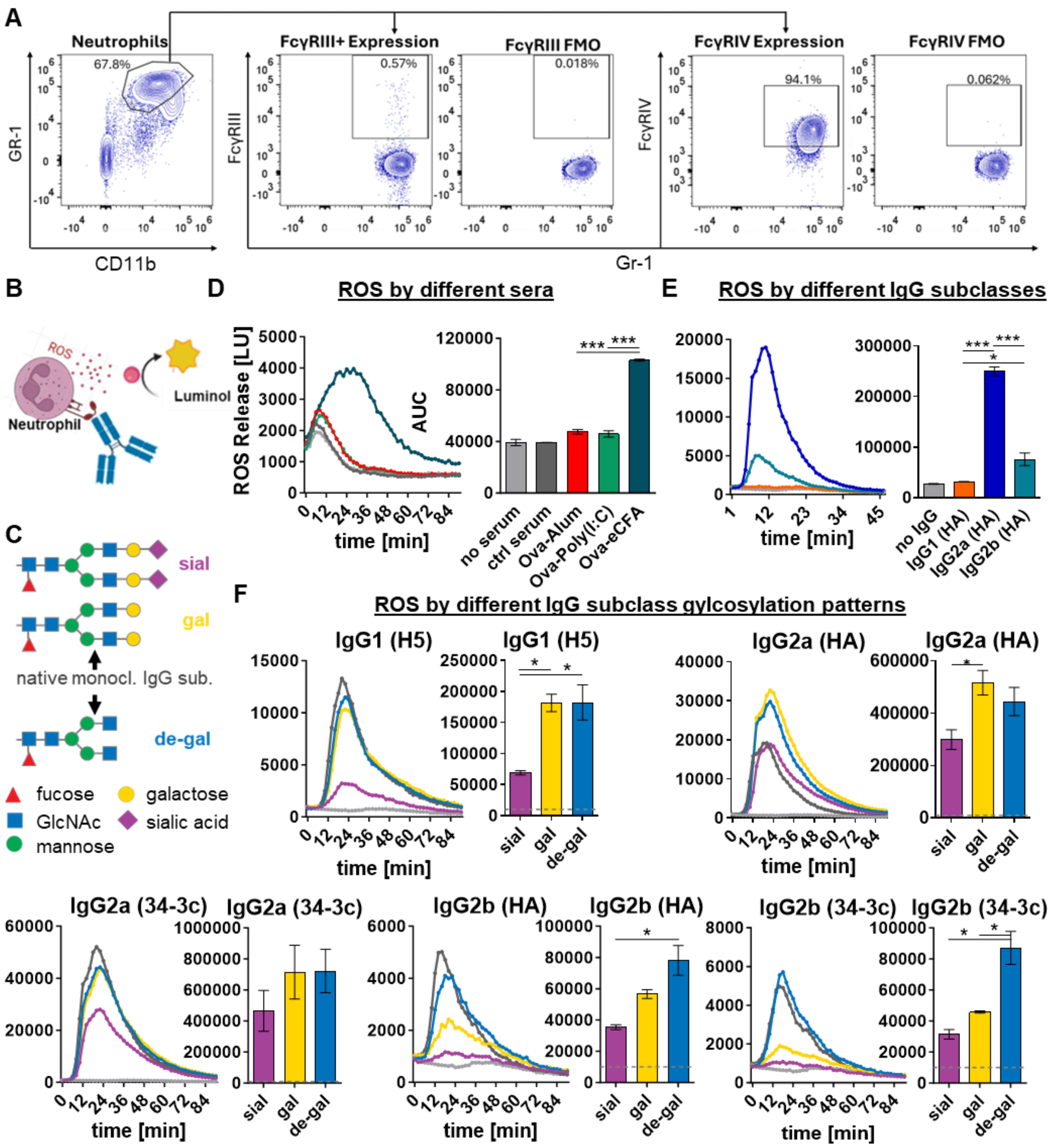

Suppl. Figure S12: Effect of vaccination-induced anti-Ova serum antibodies and glycoengineered IgG subclass monoclonal antibodies on neutrophil activation in vitro.

(A) Bone marrow neutrophils (CD11b^{+}Gr-1^{+}) from untreated C57BL/6 mice and their expression of FcγRIII and FcγRIV (compared to Fluorescence Minus One (FMO) controls) were analyzed by flow cytometry. **(B)** Schematic representation of the assay, and **(C)** targeted glycoengineered IgG patterns. **(D-F)** Release of reactive oxygen species (ROS) from neutrophils was measured in response to: (D) serum from immunized mice; (E) native monoclonal anti-TNP IgG subclass mAbs; and (F) glycoengineered (sial, gal and de-gal) anti-TNP (clones H5 and HA) and anti-erythrocyte (clones 34-C) IgG subclass mAbs. Measurements were performed in duplicate over 90 min using luminescence (LU). The area under the curve (AUC) was analyzed using GraphPad Prism. Statistics: One-way ANOVA with Tukey's multiple comparisons test. *p < 0.05, **p < 0.01, ***p < 0.001, ****p < 0.0001.

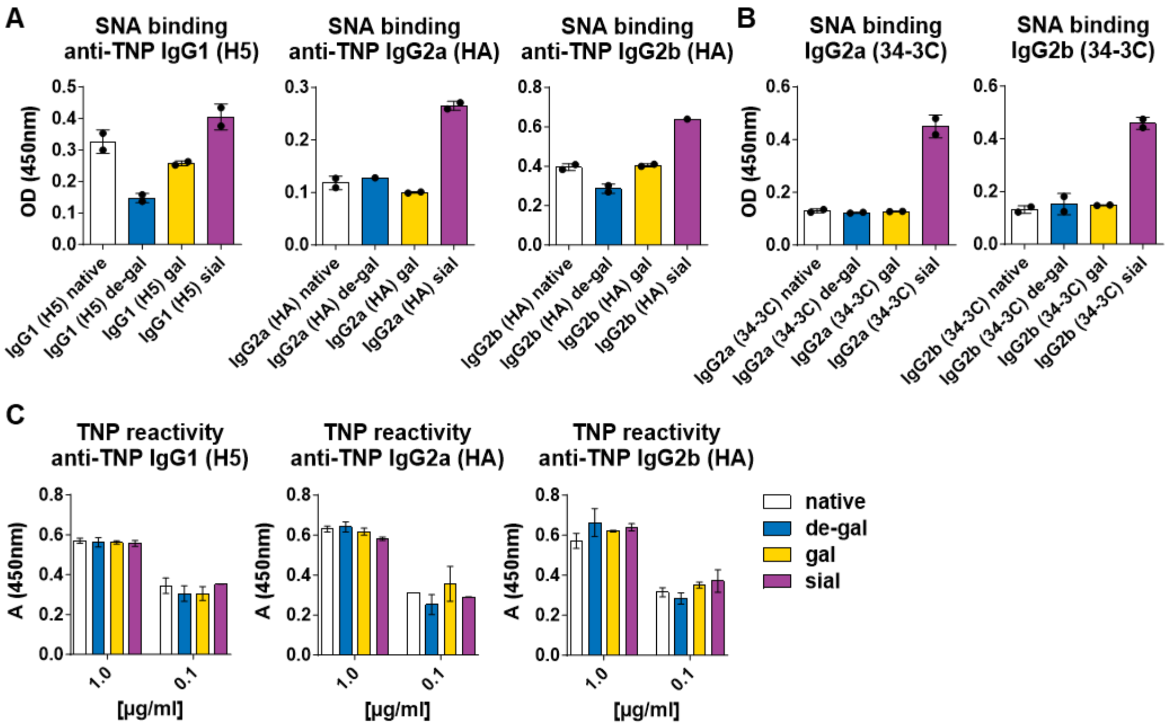

Supplementary Fig. S13: Characterization of differentially glycoengineered murine monoclonal IgG antibodies.

(A and B) Analysis of IgG sialylation using an SNA ELISA. The differentially glycoengineered (A) anti-TNP and (B) anti-erythrocyte IgG subclass mAbs, as used in Figure 2H-K and Suppl. Figure S11, were coated directly onto the plate (1 µg/ml) and sialylation was detected using SNA-HRP. (**C**) Determination of TNP binding of anti-TNP IgG subclass mAbs. Plates were coated with 10 µg/ml TNP-Ova. The differentially glycoengineered anti-TNP IgG subclass mAbs were diluted as indicated and added to the TNP-Ova-coated plate. Detection was then performed using anti-IgG-HRP (1:10,000). Each sample was applied in duplicate.

Suppl. Table S1: IgG glycoforms. Abbreviation and proposed structure of the identified IgG subclass Fc-glycoforms.

Glycoform	Structure	IgG1	IgG2c and IgG2b
G0	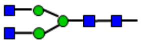	x	no
G0F	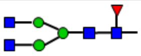	x	x
G1	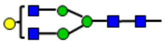	x	no
G1F	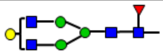	x	x
G2	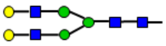	x	no
G2F	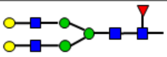	x	x
G3F	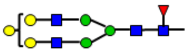	x	no
G4F	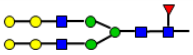	x	no
G1S1F	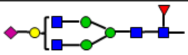	x	x
G2S1F	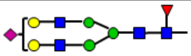	x	x
G3S1F	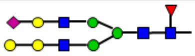	x	no
G2S2F	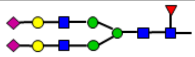	x	x

Suppl. Table S2: Description and formula for the calculation of the IgG subclass glycosylation traits.

Glycosylation trait	Description	Formula (based on Supplementary Table S1)
**fucosylation**	*N*-glycans carrying a core fucose	(G0F + G1F + G2F + G3F + G4F + G1S1F + G2S1F + G3S1F + G2S2F) / sum of all glycopeptides
**galactosylation**	*N*-glycans carrying a galactose	(G1 + G1F + G2F + G3F + G4F + G1S1F + G2S1F + G3S1F + G2S2F) / sum of all glycopeptides
**sialylation**	*N*-glycans carrying an *N*-glycolylneuraminic acid (sialic acid)	(G1S1F + G2S1F + G3S1F + G2S2F) / sum of all glycopeptides
